# Neurobehavioral phenotypes of neuronopathic mucopolysaccharidoses

**DOI:** 10.1186/s13052-018-0561-2

**Published:** 2018-11-16

**Authors:** Rita Barone, Alessandra Pellico, Annarita Pittalà, Serena Gasperini

**Affiliations:** 1 Neuropsichiatria Infantile, Dipartimento di Medicina Clinica e Sperimentale, Università di Catania, Catania, Italy; 2 Centro di Riferimento Regionale per le malattie metaboliche congenite, Policlinico, Università di Catania, Catania, Italy; 3 UOS Malattie Metaboliche Rare, Clinica Pediatrica, Fondazione MBBM, ATS Monza, Monza, Italy; 4 Neuropsichiatria Infantile, Policlinico, Università di Catania, Via S. Sofia 78, 95123 Catania, Italy

**Keywords:** Mucopolysaccharidoses, Cognition, Behavior, Assessment

## Abstract

Mucopolysaccharidoses (MPS) are a group of lysosomal multisystemic, chronic, and progressive diseases characterized by the storage of glycosaminoglycans (GAGs) that may affect the central nervous system. Neuronopathic MPS such as MPS IH, MPS II, MPS IIIA–D, and MPS VII are characterized by neurocognitive regression. In severe MPS I (MPS IH, or Hurler syndrome) initial developmental trajectory is usually unremarkable but cognitive development shows a plateau by 2 to 4 years of age and then progressively regresses with aging. Patients with neuronopathic MPS II have a plateau of cognitive and adaptive development on average by 4 to 4.5 years of age, although there is significant variability, followed by progressive neurocognitive decline. In patients with classic MPS III, developmental trajectory reaches a plateau around 3 years of age, followed by regression. Sleep disturbances and behavioral problems occur early in MPS II and III with features of externalizing disorders. Acquired autism-like behavior is often observed in children with MPS III after 4–6 years of age. Impaired social and communication abilities do occur, but MPS III children do not have restricted and repetitive interests such as in autism spectrum disorder. MPS type VII is an ultra-rare neuronopathic MPS with a wide clinical spectrum from very severe with early mortality to milder phenotypes with longer survival into adolescence and adulthood. Most patients with MPS VII have intellectual disability and severely delayed speech development, usually associated with hearing impairment. Cognitive regression in neuronopathic MPS runs parallel to a significant decrease in brain tissue volume. Assessment of the developmental profile is challenging because of low cognitive abilities, physical impairment, and behavioral disturbances. Early diagnosis is crucial as different promising treatment approaches have been extensively studied in animal MPS models and are currently being applied in clinical trials.

## Background

Mucopolysaccharidoses (MPS) are a group of chronic and progressive lysosomal storage disorders caused by the defective degradation of glycosaminoglycans (GAGs). Progressive GAG accumulation in organs and tissues leads to the development of multisystem clinical manifestations. Somatic features are variable and may include coarse face, hepatosplenomegaly, skeletal and joint abnormalities, and cardiorespiratory disease [1]. The central nervous system (CNS) is primarily affected in neuronopathic MPS I Hurler (MPS IH), II, III(A–D), and VII. Neuronopathic MPS are characterized by developmental delay and neurocognitive regression, behavioral changes and sleep disturbances, and epilepsy [1, 2]. Brain changes associated with MPS disorders include atrophic changes and abnormalities in the white matter and perivascular spaces, communicating hydrocephalus, increased intracranial pressure, and compressive myelopathy [3]. In MPS types IV and VI, cognitive development is not affected although patients may have secondary neurological complications such as hydrocephalus and cervical spine cord compression [1, 3]. The pathophysiology of neuronal damage in neuronopathic MPS is related to the storage of undegraded GAG, in particular heparan sulfate (HS), and secondary toxic products such as GM2 and GM3 gangliosides, inflammatory cytokines, and reactive oxygen species [4]. Although all neuronopathic MPS are characterized by neuronal storage of products from incomplete HS degradation, the behavioral phenotype is variable. Behavioral problems represent hallmark features of severe MPS II and MPS III [2, 5–8]. Unlike MPS II and III, children with MPS IH are generally placid, gentle, and calm, although intellectual disability does occur and progress [2, 5]. It has been hypothesized that, in patients with MPS II and III, distinct chemical moieties occurring at the ends of incompletely degraded HS molecules may interfere with neuronal function due to chemical reactions and this might be related to the occurrence of behavioral problems [9]. Patients with neuronopathic MPS I, II, III, and VII present with developmental delay, plateauing, and cognitive regression [1, 2, 5]. Hyperactive behavior is highly prevalent in neuronopathic MPS II and is considered among the early clinical markers of CNS involvement in patients with MPS II [6, 7]. MPS III patients behave as hyperactive and impulsive children and appear prone to dangerous behavior, often leading to body injuries [8]. Lack of fear and poor attention similar to a Klüver Bucy-like syndrome and autistic-like symptomatology have been reported in patients with MPS IIIA [10] and IIIB [11]. Severe sleeping disturbances, temper tantrums, easy irritability, and mood changes also occur in patients with MPS II and III [7, 8, 12]. Epilepsy has a higher prevalence in neuronopathic MPS II and MPS III, with an incidence that increases with advancing neurocognitive regression. Seizures typically present with the features of generalized tonic-clonic seizures. Absence epilepsy, tonic, focal and myoclonic seizures and nonconvulsive status epilepticus have been also reported [13, 14].

The therapy for neuronopathic MPS is aimed at correcting enzyme deficiency and/or substrate accumulation in the brain. Experimental approaches using MPS animal models have produced promising results and led to clinical trials in patients with neuronopathic MPS [15, 16]. Knowledge on the presentation and clinical course of neuronopathic MPS may be advantageous for early diagnosis and possible early intervention. In this regard, we here report on the main current topics on neurobehavioral phenotypes and summarize therapeutic advances in the cure of neuronopathic MPS.

## Developmental and behavioral patterns of MPS

### Mucopolysaccharidosis type I

Mucopolysaccharidosis type I (MPS I) is caused by a deficiency of alpha-l-iduronidase (IDUA) and storage of HS and dermatan sulfate (DS). Prevalence is estimated to be 1/100,000. Based on age at onset, and the presence or absence of cognitive decline and the severity of systemic disease, MPS I is classified as either Hurler syndrome (severe phenotype), Hurler-Scheie (intermediate phenotype), or Scheie syndrome (mild phenotype) [1]. Children with Hurler syndrome (MPS IH) develop normally in the first months of life. Slowing of development milestones occurs by 12 months of age, and global developmental delay (mental development quotient more than two standard deviations (SDs) below the mean) may be observed by 2 years of age. Patients usually have a stasis of cognitive development by 2 to 4 years of age followed by rapid regression with aging [5]. Although motor problems, hydrocephalus, visual, and hearing defects contribute to poor cognition, developmental slowness and regression occur independently and reflect primary CNS disease [5, 17, 18]. In patients with MPS IH, hematopoietic stem cell transplantation (HSCT) halts the inexorable cognitive decline [17–19]. A recent, multicenter survey in the US showed that all of 22 untransplanted children with MPS IH were in the impaired cognitive range (greater than two SDs below the mean) at 27 months of age [18]. HSCT is currently considered the treatment of choice for patients younger than 2.5 years [20]. Prospectively collected longitudinal data showed that a very young age at transplantation (< 9 months of age) was related to optimal cognitive, language, and adaptive performances in patients with MPS IH [21]. A retrospective study of 217 transplanted MPS IH patients with a median follow-up age of 9.2 years demonstrated a significant amelioration of the MPS IH clinical course after HSCT. A younger age and preservation of cognitive function at transplantation were major predictors for better cognitive outcome post-transplant [22]. In addition, a significant residual disease burden was observed in the transplanted patients with MPS IH, such as growth retardation and specific systemic problems. A normal leukocyte alpha-iduronidase enzyme level obtained post-HSCT was a predictor for better clinical outcome in most organ systems [22]. Neuropsychological assessment in transplanted patients with MPS IH showed that despite a normal full-scale IQ, patients with MPS IH after successful HSCT may have decreased attention span and information processing. Decrease in white matter integrity and reduction of corpus callosum volume are associated with defects in attention tasks in these patients [23].

There is no cognitive decline associated with attenuated MPS I Scheie (MPS IS), with the exception of patients bearing the L238Q missense mutation paired with a nonsense mutation. These patients present with late-onset brain disease as cognitive decline starts at a later age than is seen with Hurler syndrome, and psychiatric problems emerge in adolescence [24]. The extent of systemic disease burden may act as a predictor of cognitive performance in patients with attenuated MPS I. Recently, Ahmed et al. [25] demonstrated that, in attenuated MPS I Hurler/Scheie (HS) and MPS IS, somatic disease burden measured by the Physical Symptom Score (PSS) was negatively associated with cognitive level (full-Scale IQ) (*p* < 0.001), indicative that both measures reflect disease severity [25]. In addition, physical disease and sensorial defects cause functional impairment and reduce academic performances in attenuated MPS I individuals. Enzyme replacement therapy (ERT) is the first-choice therapy for patients with attenuated MPS I. A retrospective case series including 20 patients with Hurler-Scheie syndrome within nine sibships showed that, in younger siblings, somatic signs did not develop or progress when they were absent or mild at ERT initiation. Improvements in physical disease following ERT as well as interventions including hearing aids, physical therapy, and speech therapy may result in amelioration of adaptive functioning and improved quality of life for patients with attenuated MPS I [26].

Newborn screening for MPS I will benefit the identification of severely affected patients and help early treatment [27]. Before newborn screening programs are implemented, awareness of MPS I among pediatricians and primary care providers is crucial for patient identification and prompt initiation of treatment (see also the review by Donati et al. [28] in this Supplement).

In summary, patients with MPS I Hurler have developmental delay by 12 months and stagnation of cognitive development by 2 to 4 years of age followed by rapid regression with aging. Hematopoietic stem cell transplantation is the standard treatment to prevent and to halt neurocognitive regression with an earlier age of treatment associated with improved outcomes.

### Mucopolysaccharidosis type II (Hunter syndrome)

Hunter syndrome (MPS II) is an X-linked, multisystem disorder characterized by iduronate sulfatase (IDS) deficiency which results in the accumulation of DS and HS. MPS II prevalence is around 1 in 100,000 male births. Presentation in females with skewed inactivation of the X chromosome is uncommon [1]. There is high clinical variability among patients with the same mutation, suggesting epigenetic influences in the clinical expression. Systemic manifestations of MPS II include coarse face, skeletal anomalies and joint restriction, cardiac valve disease, and hepatosplenomegaly. Based on the presence or absence of progressive neurological involvement and behavioral problems, patients with MPS II are classified as neuronopathic or attenuated, respectively [5, 6]. Although there is a significant variability and the likelihood of a long-lasting developmental plateau in some patients, neuronopathic MPS II shows a plateau of cognitive and adaptive development on average at 4 to 4.5 years of age, followed by progressive neurocognitive regression [5, 6]. Neurological findings of severe MPS II include progressive impairment of gross and fine motor abilities, epilepsy, sensorineural deafness with severe language disturbance and loss of verbal language, retinal degeneration, and sleep problems [5–7]. Seizures are frequently observed most often as staring episodes, myoclonus, and tonic-clonic seizures [6, 7]. Sleeping problems can be secondary to CNS involvement, obstructive apnea, or both [29]. Sleep disturbances present as difficulty initiating or maintaining sleep. A decrease in rapid eye movement sleep duration, atypical sleep stage distribution, and frequent leg movements when falling asleep may be observed [7, 12]. A retrospective analysis of neurodevelopmental profile in 49 children with MPS II including 37 participants with neurological deterioration detected early signs and symptoms associated with neuronopathic MPS II [7]. Seven clinical markers were identified as early predictors of cognitive dysfunction: increased activity, behavior difficulties, sleep disturbance, seizure-like behavior, perseverative chewing, and an inability to achieve sphincter control. Increased activity manifested as “high-energy behavior” and inability to focus on tasks. An index of severity score was developed, with a score ≥ 3 indicating a high likelihood of developing brain disease [7]. Such findings highlight the importance of monitoring development in children with MPS II to identify early those children who may benefit from brain-targeted therapy. Somatic disease manifestations of MPS II are controlled by intravenous ERT, while this is ineffective in preventing or treating CNS involvement [30, 31]. Among brain-targeted therapy, direct delivery of ERT in the cerebrospinal fluid (CSF) has been investigated in animal models of MPS II, and a phase 1/2 study reported the safety of intrathecal (IT) administration of idursulfase formulated for IT use in patients with neuronopathic MPS II [16].

Brain changes associated with MPS II include perivascular space enlargement, white matter abnormalities, subarachnoid space enlargement, third ventricle dilatation, hydrocephalus, and brain atrophy [31, 32]. An elevated myoinositol/creatine ratio in the gray and white matter are more frequently observed in MPS II patients with neurocognitive impairment [32]. Likewise, volumetric magnetic resonance studies show significant atrophic changes in brain tissue in MPS II patients with neurological morbidity [33]. Patients with attenuated MPS II have normal cognitive development or mild impairment [5, 6]. Despite a normal IQ, attenuated MPS II patients treated with ERT may have a decreased attention span and impaired visual processing related to decreased volumes of white matter and corpus callosum [34].

In summary, although there is a significant variability in patients with neuronopathic MPS II, cognitive development reaches a plateau on average at 4 to 4.5 years of age, followed by progressive regression. Behavioral and sleep problems represent early clinical markers of CNS involvement in patients with neuronopathic MPS II. Emerging promising treatments for neuronopathic MPS II such as IT-ERT are being evaluated.

### Mucopolysaccharidosis type III (Sanfilippo syndrome)

Sanfilippo syndrome (MPS III) includes four autosomal recessive disorders (MPS III A–D) caused by deficiency of different lysosomal enzymes required for the degradation of HS. MPS III are the most frequent among MPS disorders with an overall prevalence of 1.5–1.9 per 100,000 live births [1]. MPS III are neurodegenerative disorders characterized by regression of intellectual and motor abilities, behavioral problems, and dementia, with death usually in the second decade of life [2]. Developmental delay and, in particular, speech delay are common presenting features of MPS III. Patients with the classic severe phenotypes have developmental plateau at around 3 years of age followed by rapid regression of language, cognition, and adaptive functions, with longer preservation of motor abilities [5, 8, 35–39]. In addition to the severe phenotype, there are patients with attenuated MPS III showing a slowly progressing course with long periods of stable intellectual disability and gradual cognitive decline even in adulthood [37, 40, 41]. A prospective study of MPS IIIA classified two groups of patients as rapid and slow progressive, respectively, differing in their natural histories of cognitive and brain changes [39]. Children with rapid progression usually presented with a mean loss of –14.6 DQ points per year at an age below 6 years. On the other hand, patients with slow progression had a mean DQ reduction of –3.7 points per year. Cognitive decline ran parallel to brain atrophy and was related to a consistent decrease in cortical gray matter volume [39]. Neuropsychiatric abnormalities in MPS III are classified according to a triphasic model [8, 35, 42]. In the first 1 to 2 years, patients have a developmental delay, in particular for speech abilities (the beginning phase). Almost half of patients will never be toilet trained. At an average of 2 to 4 years of age (the middle phase), behavioral abnormalities become evident with increasingly frequent and severe temper tantrums. Patients have anxiety symptoms with panic attacks when they are in an unfamiliar environment and display phobias. This is followed by hyperactivity and a decreased attention span and impulsive behavior. The late phase of disease starts at around 10 years and it is characterized by a decrease in challenging behavior and increases in motor difficulties, spasticity, loss of balance, and feeding disturbances. Children with MPS III have significantly more behavioral problems relating to hyperactivity, orality, body movements, and inattention compared with matched controls with intellectual disability [42]. Epilepsy including generalized tonic-clonic seizures, tonic, focal and myoclonic seizures, and nonconvulsive status-epilepticus may occur in the later stages of the disease [8, 13, 14]. Recently the Sanfilippo Behavior Rating Scale (SBRS) has been developed to quantify and to monitor behavioral changes in Sanfilippo syndrome. The scale includes four abnormality clusters—movement, lack of fear, social/emotional, and executive dysfunction—and may be helpful for measuring behavioral changes that accompany disease progression [43].

Sleep alterations are an almost constant feature of MPS III. Sleep disturbances have been characterized by parental sleep questionnaires [44] and objectively recorded by polysomnography [45] and actigraphy [46, 47]. Patients with MPS III have significant longer sleep onset latencies and problems with failing to fall asleep and greater daytime sleep compared with controls. Sleep problems result in very frequent nocturnal waking and wandering, with personal and familial distress [12, 44, 46]. MPS III mice show modifications in the circadian rhythm that correlate with lysosomal storage and changes in circadian neuropeptides in the suprachiasmatic nucleus that is the main biological pacemaker in mammals [48]. Patients with MPS III show significantly increased fragmentation of circadian rhythm and abnormal endogenous melatonin concentrations [47]. Exogenous melatonin administration is effective in most MPS III patients [8, 12, 44]. Around the age of 4 years, MPS III patients may show acquired symptoms of autism spectrum disorder (ASD) such as impaired social reciprocity and communication. Unlike children with idiopathic ASD, social and communicative symptoms are evident later than 3 years of age and, apart from hyperorality, MPS III children do not present with restricted interests or repetitive behavior [49, 50]. The diagnosis of MPS III may be overlooked because of the lack of early somatic signs associated with mucopolysaccharide storage disorders [8]. Moreover, children with MPS III may be initially misdiagnosed with ASD and/or other neurodevelopmental disorders (i.e., developmental delay, language delay, attention deficit disorder with hyperactivity) because clinicians tend to focus on neurodevelopmental delay and behavioral problems that are prominent presenting features [51]. Since typically developing children have a rapid development in the first years of life, developmental plateauing at this time and/or the occurrence of behavioral problems and sleep disturbances should prompt diagnostic analyses for underlying diseases such as Sanfilippo disease even in the absence of overt somatic MPS features. Different approaches to treat brain disease such as phase 1/2 clinical trials using IT-ERT or gene therapy have been performed in patients with MPS IIIA and IIIB [16].

In summary, patients with MPS III and rapidly progressing phenotypes have a development plateau around 3 years of age followed by progressive decline. Patients with attenuated phenotypes may have a long period of stable intellectual disability before regression. MPS III may be initially misdiagnosed because of the lack of early somatic signs associated with MPS. Emerging promising treatments for brain disease in MPS III have been developed and are being used in phase 1/2 clinical trials.

### Mucopolysaccharidosis type VII (Sly syndrome)

Sly syndrome or MPS VII is a very rare neuronopathic MPS characterized by a deficiency of the lysosomal enzyme β-glucuronidase. MPS VII features include coarse face, short stature, skeletal disease, joint stiffness, hepatosplenomegaly, and cardiorespiratory disease. CNS involvement is prominent in patients with MPS VII. Neurological features include global developmental delay, speech delay, intellectual disability of variable degree, sensorineural deafness, hydrocephalus, and spinal cord compression [1, 52]. The disease shows a wide range of clinical variability. Based on their phenotype, patients with MPS VII have been recently classified into three different groups. Nonimmune neonatal hydrops fetalis patients (the first group) have severe multisystemic disease and survive a few months. Patients with infantile or adolescent forms with or without history of hydrops fetalis (the second and third groups, respectively) vary from mild to moderate or severe phenotypes independent of the occurrence of neonatal hydrops fetalis [53]. Somatic features of patients with MPS VII resemble those of MPS I and MPS II; however, the frequent occurrence of nonimmune hydrops fetalis (almost 40% of patients) is a distinguishing clinical feature [54]. Global developmental delay and intellectual disability (mild, moderate to severe), limited vocabulary, and speech disturbance are common. Hearing impairment is frequent and likely contributes to speech disturbance [53, 55]. Patients usually require special education. A survey on the clinical course of MPS VII reported that 17% of 46 patients with infantile or adolescent forms were able to perform daily activities by themselves, while most patients required assistance. Almost 50% of patients could walk without support, although most patients lost this ability. Patients with mild or moderate early manifestations tended to deteriorate more slowly than patients with more severe manifestations [53]. Treatment approaches for MPS VII including bone marrow transplantation (BMT), ERT, and gene therapy are very promising [16, 53, 56, 57]. Information on patients with MPS VII that underwent BMT at variable ages and disease stages suggest that engrafted BMT may slow or prevent neurological deterioration [19, 53, 56]. Investigational therapy with recombinant human β-glucuronidase (rhGUS) over 24 weeks in a 12-year-old boy with advanced stage MPS VII was associated with a reduction in urinary GAG, normalization of liver and spleen sizes, improvement of pulmonary function, and increased activity level and quality of life [57]. Clinical trials of ERT on patients with MPS VII are underway.

In summary, patients with MPS VII have a prominent CNS involvement and somatic features resembling those of MPS I and MPS II. Novel promising treatments for MPS VII such as intravenous ERT are being employed in clinical trials.

## Cognitive assessment tools in children with MPS

Cognitive measurements in MPS are particularly important for natural history studies and are increasingly required to compare efficacy across clinical trials. A definition of cognitive outcome measures is challenging due to the rarity and specific features of MPS patients. Assessment protocols need to be uniform to make data comparable across ages. The formal assessment of cognitive functions in patients with MPS II and III poses several major challenges because of physical and behavioral limitations in this population. Detailed information on neurocognitive assessment in patients with MPS have been reported [58–62]. Cognitive endpoints for therapy development were discussed in a consensus paper [63]. General issues include the use of age-equivalent measures, especially at younger ages, to evaluate changes over time as well as the need for precise, simple measurements, to control the testing environment, and to use the appropriate test for the estimated developmental age [37, 58–63]. The main cognitive assessment tests employed in patients with neuronopathic MPS according to age are reported in (Table 1). The Griffiths’ Mental Development Scales-II, the Bayley Scales of Infant Development (BSID)-III or the Kaufman Assessment Battery for Children (KABC)-II have been used in children with MPS under 3 years of age or those with an estimated developmental age ≤ 3 years [37, 39, 58, 61]. The BSID-III age-equivalent and developmental quotients have been suggested in patients with MPS I [18] and MPS III [58]. The disadvantage of BSID-III is its limited age range that requires a transition to different tools over time. The Mullen Scales of Early Learning (MSEL), mostly used in the US, are applicable from 0 to 68 months of age and have been proven to measure disease-related changes in MPS II [6], III [38], and in MPS I patients post-HSCT [18, 21]. The Wechsler scales (Wechsler Preschool and Primary Scale of Intelligence (WPPSI), Wechsler Intelligence Scale for Children (WISC), and Wechsler Adult Intelligence Scale (WAIS)) are structurally similar and, by covering a wide range of ages, have been used in MPS-I post-HSCT and in other MPS over the age of 3 years whenever possible [61–63]. MPS patients have limitations due to motor disability, visual and hearing defects, and behavioral problems. The Wechsler Abbreviated Scale of Intelligence (WASI)-II is short and does not penalize patients with motor difficulties or slow processing speed; it has already been used in patients with MPS-I [23] and II [34]. The Wechsler scales demanding fine motor skills, sustained attention, and processing speed are not feasible in patients with MPS II and III with low cognitive levels and behavioral concerns. The Vineland Adaptive Behavior Scales (VABS)-II is parent observation of adaptive behavior administered by interview that includes communication, daily living skills, socialization, and motor skills. The VABS are recommended for measuring adaptive behavior in patients with MPS at all ages [62, 63] and allow parent-reported age-equivalent scores to be obtained and compared with those of the cognitive measures [58]. In conclusion, cognitive assessment tools in patients with neuronopathic MPS should take into account the proper clinical features of this population. The assessment of these children requires a quiet environment facilitating concentration; to obtain this, the child is rested and calm during the assessment, and this should be performed preferably as the first test after admission to hospital before other stressful examinations [38].

**Table 1:** Neurocognitive tests employed in patients with neuronopathic MPS according to age

	Tests employed			References
Age ≤ 36 months	BSID-III	MSEL	GMDS-III	
Range^a^	0–42 months	0–68 months	0–72 months	
Natural history, MPS characteristic studies	MPS I, II, III	MPS I, II, III	MPS III	[6, 19, 37, 38, 60]
Treatment outcome	MPS I (HSCT), MPS II (ERT); MPS IIIA (IT-ERT)	MPS I (HSCT)	MPS I (HSCT, ERT), MPS II (HSCT, ERT), MPS III (HSCT)	[18, 21, 29, 60]
Age > 36 months, ≤ 6 years	KABC-II	Leiter-III	WPPSI-IV	
Range^a^	3–18 years	3–75 years	2.6–7.7 years	
Natural history, MPS characteristic studies	MPS III	–	MPS I, II	[29, 33, 48, 60, 61]
Treatment outcome	–	MPS I ((HSCT), MPS III (HSCT)	MPS I (HSCT)	[60–62]
Age > 6 years and adulthood	WISC-IV	WAIS-IV	WASI	
Range^a^	6.0–16.11 years	16–90 years	6–89 years	
Natural history, MPS characteristic studies	MPS II, III	MPS I, II, III	MPS I, II	[18, 31, 40, 60–62]
Treatment outcome	MPS I (HSCT)	MPS I (HSCT)	MPS I (HSCT)	[23, 25, 60–62]

*BSID* Bayley Scales of Infant Development, *ERT* enzyme replacement therapy, *GMDS* Griffiths Mental Development Scales, *HSCT* hematopoietic stem cell transplantation, *IT* intrathecal, *KABC* Kaufman Assessment Battery for Children, *MPS* mucopolysaccharidosis, *MSEL* Mullen Scales of Early Learning, *WAIS* Wechsler Adult Intelligence Scale, *WASI* Wechsler Abbreviated Scale of Intelligence, *WISC* Wechsler Intelligence Scale for Children, *WPPSI* Wechsler Preschool and Primary Scale of Intelligence

^a^ Range of age referred according to manual

In summary, cognitive function should be measured by age-equivalent scores in neuronopathic MPS patients. Assessment tools should be selected taking into account the physical, sensorial, and behavioral limitations present in MPS patients.

## Principles of treatment of neuronopathic MPS

Currently, different brain-targeted approaches are in preclinical and clinical development with the aim of preventing or halting primary CNS involvement of MPS. HSCT aims to deliver donor stem cells that produce the deficient enzyme in lysosomal storage disorders [19]. HSCT is a consolidated and effective therapy to treat brain disease manifestations in MPS IH [17]. The experience of HSCT in the treatment of other neuronopathic MPS is limited, although it appears to have a modest impact on neurological disease in patients with MPS II and III treated in the early stages of the disease [16, 64, 65]. At this time, intravenous ERT represents an effective cure for systemic manifestations of MPS I, II, IVA, and VI, leading to improvement in the disease features and patient quality of life (see also the review by Concolino et al in this Supplement [66]). The efficacy of investigational therapy with intravenous recombinant human β-glucuronidase (rhGUS) over 24 weeks was shown in a patient with advanced stage MPS VII [57]. The selective permeability of the blood–brain barrier (BBB) hampers the effects of systemic intravenous ERT on the CNS due to the limitations of large enzyme molecules crossing the BBB and preventing neurodegeneration [16]. New promising treatments for neuronopathic MPS disorders include direct delivery of ERT into the CNS through either intracerebroventricular injection or IT injection via an IT drug delivery device (IDDD) into the lumbar spine or subarachnoid space at the cisterna magna [67]. Direct delivery of ERT in the CSF has been extensively tested in animal models of neuronopathic MPS I [68], II [69], and III [70]. Phase 1/2 safety trials of IT-ERT in patients with MPS I [71], MPS II [72], MPS IIIA [73], and IIIB [74] have been published. Recent advances in genetic therapies including development of gene vectors and delivery routes are very promising for treating the neurologic manifestations of MPS [75]. A phase 1/2 study evaluating the effects of intracerebral injection of adeno-associated viral vector rh.10-SGSH-IRES-SUMF1 and immunosuppressive treatment in four children with MPS IIIA showed a good safety profile and suggested moderate amelioration in behavior, attention, and sleep in the first year after surgery [76]. Recently, a phase 1/2 clinical trial was conducted in four children with MPS IIIB aged 20, 26, 30, and 53 months. A recombinant adeno-associated viral vector serotype 2/5 (rAAV2/5) encoding human α-*N*-acetylglucosaminidase (NAGLU) plus immunosuppressive therapy was used. The study showed good safety and tolerability and induced sustained enzyme production in the brain. There was an improvement in neurocognitive progression compared with the natural history of MPS III, although a longer follow-up was envisaged to assess safety outcomes and persistency of improved cognitive development [77]. Additional promising approaches for the treatment of CNS disease in MPS include enzyme-fusion protein technology (Trojan horse strategy), substrate reduction therapy, chaperone molecules, and the use of nanoparticle-delivered therapy [16, 78, 79] (see also the reviews by Fecarotta et al. [80] and Fraldi et al. [81] in this Supplement). In addition to the treatment of primary CNS disease, children with MPS benefit from adjunctive therapies such as speech/language therapy, and occupational and physical therapy. Practical interventions, environmental changes, and behavioral interventions can be helpful in managing behavioral and sleep problems [8, 12, 44]. A number of antiepileptic drugs (AED) have been used to treat epilepsy in patients with MPS. Monotherapy is generally effective, but less frequently two AED may be required for controlling seizures [13, 14]. The experience with behavior-modifying medications is most often empirical [12]. In patients with MPS, the risk of side effects is high for antipsychotics along with the occurrence of paradoxical effects of anti-anxiety drugs, such as over-activity or over-sedation [8, 12]. Melatonin use is considered effective in the management of sleep disturbances [44], consistent with proven altered circadian rhythm in children with MPS III [46, 47].

## Conclusions

Neurocognitive evaluation in children with neuronopathic MPS may be challenging because of possible very low cognitive functions and behavioral disturbances, and sensorial and physical impairments. A careful preliminary clinical evaluation should be performed to recognize or exclude hearing loss (acute or chronic, conductive or sensorineural), difficulties in fine hand movements due to bone deformities and joint stiffness, and other orthopedic disturbances that impair activities requiring strength or balancing. Accurate assessment is required in these children to monitor early disease progression and outcome, particularly in view of the novel therapeutic approaches available. Knowledge of the neurobehavioral phenotypes of MPS may be critical for recognition, early diagnosis, and intervention programming and monitoring. It can be helpful for families in order to plan the future care of their child.

## Abbreviations

AED: Antiepileptic drugs
ASD: Autism spectrum disorder
BBB: Blood–brain barrier
BMT: Bone marrow transplantation
BSID: Bayley Scales of Infant Development
CNS: Central nervous system
CSF: Cerebrospinal fluid
DS: Dermatan sulfate
ERT: Enzyme replacement therapy
GAG: Glycosaminoglycan
GMDS: Griffiths Mental Development Scales
HS: Heparan sulfate
HSCT: Hematopoietic stem cell transplantation
IT: Intrathecal
KABC: Kaufman Assessment Battery for Children
MPS: Mucopolysaccharidosi(e)s
MSEL: Mullen Scales of Early Learning
VABS: Vineland Adaptive Behavior Scales
WAIS: Wechsler Adult Intelligence Scale
WASI: Wechsler Abbreviated Scale of Intelligence
WISC: Wechsler Intelligence Scale for Children
WPPSI: Wechsler Preschool and Primary Scale of Intelligence
